# Pharmacological Inhibition of P-Rex1/Rac1 Axis Blocked Angiotensin II-Induced Cardiac Fibrosis

**DOI:** 10.1007/s10557-023-07442-3

**Published:** 2023-03-09

**Authors:** Jianyuan Pan, Ming Liu, Huimin Su, Hao Hu, Hongwu Chen, Likun Ma

**Affiliations:** Department of Cardiology, The First Affiliated Hospital of USTC, Division of Life Sciences and Medicine, University of Science and Technology of China, No.17 Lujiang Road, Hefei, Anhui 230001 People’s Republic of China

**Keywords:** P-Rex1, Cardiac fibroblast, Fibrosis, Reactive oxygen species, Rac

## Abstract

**Purpose:**

Phosphatidylinositol-3,4,5-trisphosphate-dependent Rac exchange factor-1 (P-Rex1), as one of the members of Rac-GEFs, has been proven to play a critical role in cancer progression and metastasis. Nonetheless, its role in cardiac fibrosis remains elusive. In the present study, we aimed to investigate whether and how the P-Rex1 mediates AngII-induced cardiac fibrosis.

**Method:**

A cardiac fibrosis mouse model was established by chronic AngII perfusion. The heart structure, function, pathological changes of myocardial tissues, oxidative stress, and cardiac fibrotic protein expression were determined in an AngII induced mouse model. To provide a molecular mechanism for P-Rex1 involvement in cardiac fibrosis, a specific inhibitor or siRNA was used to block P-Rex1, and target the relationship between Rac1-GTPase and its downstream effector.

**Results:**

Blocking P-Rex1 showed down-regulation of its downstream effectors such as the profibrotic transcriptional regulator Paks, ERK1/2, and ROS generation. Intervention treatment with P-Rex1 inhibitor 1A-116 ameliorated AngII-induced abnormalities in heart structure and function. Moreover, pharmacological inhibition of the P-Rex1/Rac1 axis showed a protective effect in AngII-induced cardiac fibrosis through the down-regulation of collagen1, CTGF, and α-SMA expression.

**Conclusion:**

Our findings demonstrated for the first time that P-Rex1 was an essential signaling mediator in CFs activation and subsequent cardiac fibrosis, and 1A-116 could be a potential pharmacological development drug.

**Supplementary Information:**

The online version contains supplementary material available at 10.1007/s10557-023-07442-3.

## Introduction

Pathological cardiac hypertrophy, characterized by myocardial enlargement and cardiac fibrosis, is associated with the long-term elevation of angiotensin II (AngII) and aldosterone levels [1]. Robust clinical evidence suggests a strong relationship between cardiac fibrosis and adverse outcomes in patients with cardiac disease [2]. In many cases, fibroblasts transdifferentiation into myofibroblasts and/or activated fibroblasts is considered to be one of the key cellular events in response to myocardial infarction (MI) [3], myocarditis [4], volume overload [5], and alcoholic cardiomyopathy [6]. However, understanding the molecular mechanisms underlying cardiac fibrosis and the methods of preventing cardiac fibrosis remain limited [7]. Therefore, it is of the utmost significance to elucidate the mechanisms and further develop novel drugs for combating myocardial fibrosis.

Past studies have demonstrated that Rac1 activation played a key role in biological processes such as cell migration, survival, and proliferation [8]. Accumulating evidence indicates that activation and/or overexpression of Rac1 was associated with ECM expression and cardiac fibrosis [9]. Interestingly, hyperactivated Rac1 is mainly due to alterations in its regulatory protein. Activation of guanine nucleotide exchange factors (GEFs) is the most common mechanism for signal-induced Rac-GTPase activation [10]. This activation was commonly induced by G protein-coupled receptors (GPCRs), such as AngII or Endothelian (ET-1) [11].

Phosphatidylinositol-3,4,5-trisphosphate-dependent Rac exchange factor-1 (P-Rex1), as one of the members of Rac-GEFs [12], has been proven to play a critical role in cancer progression and metastasis through a feedback loop of Rac1 activation [13]. Nonetheless, the role of Rac-GEF P-Rex1 in cardiac fibrosis remains elusive.

In the present study, we first reported that P-Rex1 was up-regulated in an AngII induced cardiac remodeling model. In addition, P-Rex1 was an essential mediator in AngII-driven Rac1 activation and responded to cardiac fibrosis. Signals from the activation of GPCRs converge on P-Rex1 to mediate Rac1 activation. Based on this model, intervention treatment with 1A-116 was used to investigate the effect of P-Rex1 specific inhibitor on preventing AngII-induced cardiac remodeling, and the role of P-Rex1/Rac1 pathway and ROS production in this process. Our findings suggested that P-Rex1 represents a potential therapeutic target to prevent AngII-induced cardiac fibrosis through the downregulation of Rac1 activation and ROS production.

## Methods

### Animals and Animal Models

Male C57BL/6 mice aged 9–10 weeks with a weight of 24.5–29.5 g were housed in the pathogen-free mouse room. The mice were subjected to chronic angiotensin II (1.0 mg/kg/ day) via osmotic minipumps to establish a cardiac fibrosis model. P-Rex1 expression in the whole heart tissue was examined on days 3, 7, 14, and 28 (*n* = 6 for each group). In addition, angiotensin II-induced cardiac remodeling mice received daily intraperitoneal injections of 50 μl PBS, 5 mg/kg mouse 1A-116 (Cat. No. 6701/10, from Tocris company), which is a P-Rex1 specific inhibitor and has been proved by Cardama (*n* = 6 for each group). At 4 weeks, the hearts were dissected and weighed to calculate the heart weight/body weight (HW/BW) and heart weight/tibia length (HW/TL) ratios. All the experimental procedures were performed following the institutional guidelines of animal care and were approved by the ethics committee of the first affiliated hospital of USTC University.

### Culture of Cardiac Fibroblasts

The hearts were taken from one to three days old neonatal rats and cardiac fibroblasts (CFs) were isolated. After 24–48 hours of growth, CFs would be passaged in a ratio of 1:3 and used for the indicated experiments. CFs were assigned to different groups, including PBS group, AngII group, siRNA group, 1A-116 treatment group, AngII+1A-116 group, AngII+siRNA group, respectively. CFs were incubated with angiotensin II human (10^–5^ M, Sigma, USA) for the indicated time.

### Active Rac1 Pull-Down

Approximately, 70–80% of the confluent of NRCFs was lysed with lysis buffer and then the pellet was collected after centrifuge (14,000×g for 5 min at 4 °C). For the detection of active Rac1-GTP, the Rac1 Activation Magnetic Beads Pull-down Assay (Merck, 17-10393, Darmstadt, Germany) was applied according to the manufacturer’s instructions. Then, the samples were applied to a polyacrylamide gel along with the beads and transferred onto membranes. First, we use ponceau S in 0.1% acetic acid stain for total protein normalization. Then, we washed ponceau S out and the membranes were blocked and then incubated with anti-Rac1 (ab282581, Abcam, 1:1000) antibodies at 4 °C overnight. The secondary antibodies were incubated at room temperature for 1 h. The blots were visualized using a High-sig ECL western blotting substrate (Tanon, Shanghai, China).

### Western Blot Analysis

The whole heart tissue or CFs was lysed in RIPA lysis buffer, and the total protein was extracted and detected with a BCA Protein Assay Kit. Approximately 20 μg of total protein was separated by electrophoresis on 6% to 15% SDS-polyacrylamide gels. After electrophoresis, the samples were transferred to immobilon-FL PVDF membranes. The membranes were blocked and then incubated with anti-P-Rex1 (ab175431, Abcam, 1:1000), anti-Rac1 (ab282581, Abcam, 1:1000), anti-CTGF (sc-365970, Santa Cruz, 1:800), anti-α-SMA (sc-53142, Santa Cruz, 1:1000), anti-collagen1 (sc-59954, Santa Cruz, 1:500), anti-Paks (2604, Cell signaling, 1:1000), anti-phospho-Paks (2606, Cell signaling, 1:1000), anti-total ERK1/2 (4695, Cell signaling, 1:1000), anti-phospho-ERK1/2 (4370, Cell signaling, 1:1000), anti-P38 (9212, Cell signaling, 1:1000), anti-phospho-P38 (4511, Cell signaling, 1:1000), and anti-GAPDH (5174, Cell signaling, 1:1000) antibodies at 4 °C overnight. The secondary antibodies were incubated at room temperature for 1 h. The blots were visualized using High-sig ECL western blotting substrate (Tanon, Shanghai, China).

### Migration and Proliferation Assay

CFs were seeded in a 24-well plate or cultured in the Transwell unit. CFs were starved overnight and cultured for 24 h with inhibitor or siRNA for P-rex1, then stimulated with AngII. The fibroblasts would be incubated with Roti©-ImmunoBlock at RT, the Ki67 antibody (12075, Cell signaling, 1:200) was added overnight at 4 °C. Then the fibroblasts were washed thrice with PBS and incubated with the respective secondary antibody, DAPI, and TRITC-phalloidin for 1 h at RT. Images were taken with a fluorescence microscope to count the ratio of Ki67-positive cells to DAPI-stained nuclei. The invasive cells were counted as the number of migrated fibroblasts under Zeiss microscope.

### Measurement of Nox Activity

The Nox activity in the CFs was measured by enhanced lucigenin chemiluminescence. The debris was removed by centrifugation at 12,000×g for 10 min at 4 °C and the supernatant was collected; 100 μM of NADPH was mixed with the supernatant and reacted with Nox to generate superoxide anions. The reaction of lucigenin (5 μM) with superoxide anions showed light emission, and the values represented the Nox activity as the mean light units (MLU) per minute per milligram of protein.

### RNA Isolation and Real-Time RT-PCR

The total mRNA was extracted from whole cardiac tissues using TRIZOL reagent, and cDNA was synthesized according to the manufacturer’s instructions. The primer sequences were for P-Rex1: 5’-GGCATTCCTGCATCGCATC-3’, reverse primer: 5’- CGGGTGTAAACAATACTCCAAGG-3’; α-SMA: 5’-ACTCTCTTCCAGCCATCTTTCA-3’, 5’-ATAGGTGGTTTC GTGGATGC-3’; collagen I: 5’-CATGTTCAGCTTTGTGGACCT-3’, 5’-GCAGCTGACTTCAGGGATGT-3’; CTGF: 5’-TGTGTGATGAGCCCAAGGAC-3’, 5’-AGTTGGCTCGCATCATAGTTG-3’; IL-6:5’-AGTTGCCTTCTTGGGACTGA-3’, 5’-TCCACGATTTCCCAGAGAAC-3’. The relative mRNA expression of P-Rex1, CTGF, collagen 1a1, IL-6, and α-SMA were analyzed by RT-PCR and normalized to the expression of the PPIA housekeeping gene.

### Echocardiography

Echocardiographic images were obtained using an ultrasound system with a 21-MHz probe under isoflurane anesthesia (2.0–3.0%). When the heart rate was around 400 bpm, we measured the left ventricle (LV), the heart rate (HR), LV end-systolic diameter (LVESD), LV end-diastolic diameter (LVEDD), LV posterior wall thickness (LVPWD), end-diastolic ventricular septal thickness (IVSD), ejection fraction (EF), and fractional shortening (FS) using two-dimensional parasternal long-axis and short-axis views at the level of the papillary muscle.

### Histological Analysis

After hearts were fixed with 4% neutral paraformaldehyde for 7 days, cardiac sections (5 μm) of left ventricular sections were cut from the same location of each heart and examined by Hematoxylin and eosin (H&E) staining and Sirius red staining (for morphometric analysis, photographs were observed under × 400 magnification with Carl Zeiss GmbH, Oberkochen, Germany). Interstitial fibrosis and the cardiac fibrosis volume fraction were calculated as the ratio of the stained fibrotic area to the total myocardial area (the mean percentages of collagen fiber occupied parts in LV (%/mm^2^ of field)).

### Statistical Analyses

Data from three to five independent experiments were shown as the mean±SD and processed using Prism 7 (GraphPad Software Incorporated, USA). One way ANOVA with Dunett-t post hoc correction was used to compared between the individual groups and the control group. We show a representative immunoblot in the main figures, changes in protein/phosphorylation levels from three independent experiments were semi-quantified by densitometry and were shown as ratios or fold changes from different groups. A *p*-value < 0.05 was considered significant.

## Results

### AngII Perfusion Induces Cardiac Fibrosis and Increases P-Rex1 Expression

**Fig. 1:**
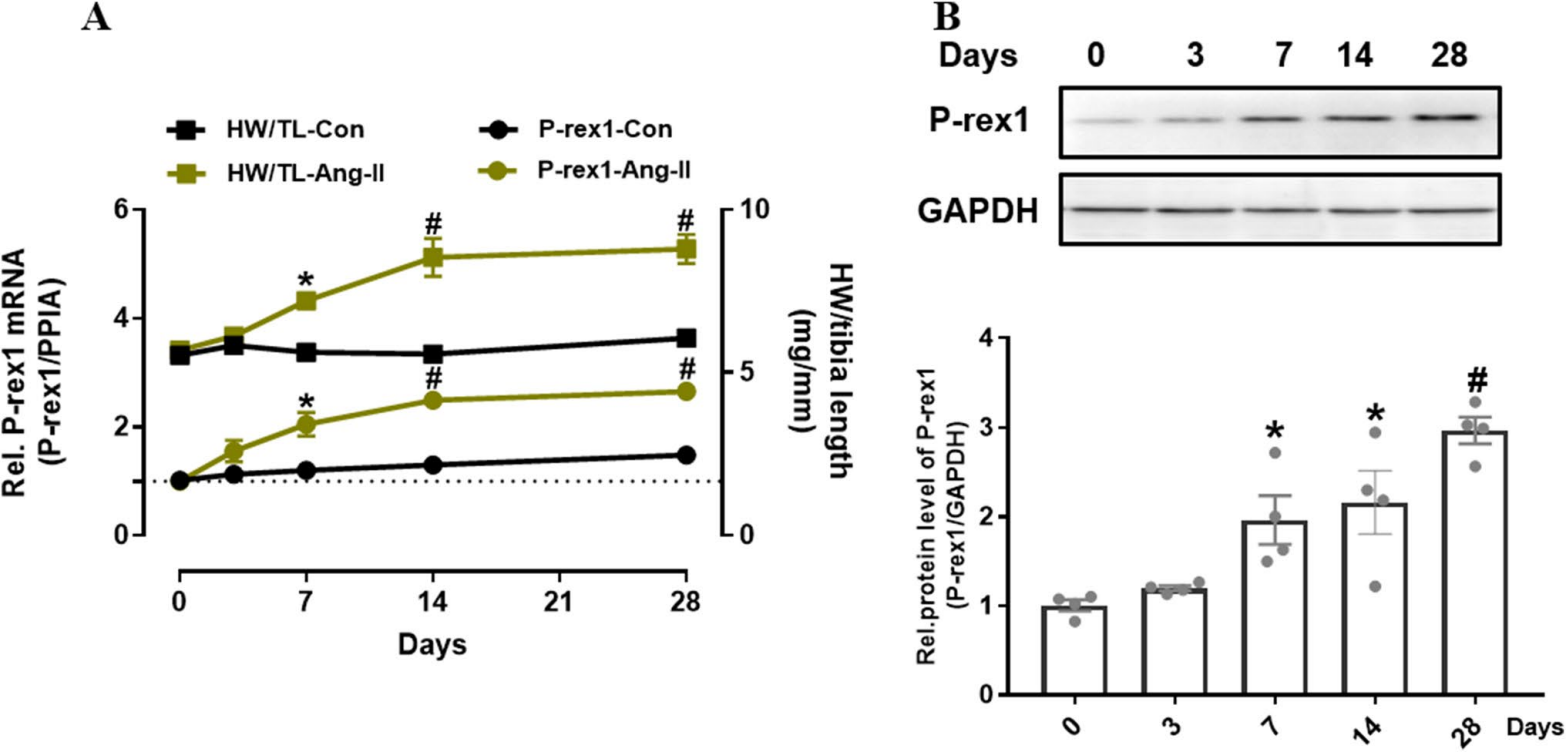
P-Rex1 expression was upregulated in mouse cardiac tissue after chronic AngII perfusion. **A** Time-dependent heart weight to tibia length ratio and P-Rex1 mRNA level (qPCR, whole heart tissue) after 3, 7, 14, and 28 days of AngII perfusion. **B** P-Rex1 expression determined by immunoblot and quantified after chronic AngII perfusion. GAPDH was a loading control. Values are given relative to saline controls, Data are mean±SD for *n* = 6 mice. ^*^P < 0.05, ^#^*P* < 0.01 versus saline control

To demonstrate the critical role of P-Rex1 in cardiac fibrosis and to validate P-Rex1 as a potential therapeutic target, we first tested the expression of P-Rex1 in a progressive model of cardiac fibrosis. C57BL/6 mice received AngII perfusion (1 mg·kg^−1^·d^−1^) via osmotic pumps for 28 days. The ratio of HW/TL in AngII-treated mice was increased compared with the control group (Fig. 1A). A time-dependent upregulation was observed in P-Rex1 mRNA expression, which paralleled the increase in the ratio of HW/TL (Fig. 1A). Western blot was used to examine the P-Rex1 protein expression, which also was lysed from whole heart tissue. The results of WB and quantitative analysis showed that the P-Rex1 had a significant upregulation after chronic AngII perfusion (Fig. 1B). Thus, this evidence proved that the cardiac remodeling mouse model was successfully established. Moreover, the upregulation of P-Rex1 indicated that it may play a key role in cardiac fibrosis progression in response to AngII perfusion.

### P-Rex1 is an Essential Mediator of Rac1 Activation in Response to AngII

**Fig. 2:**
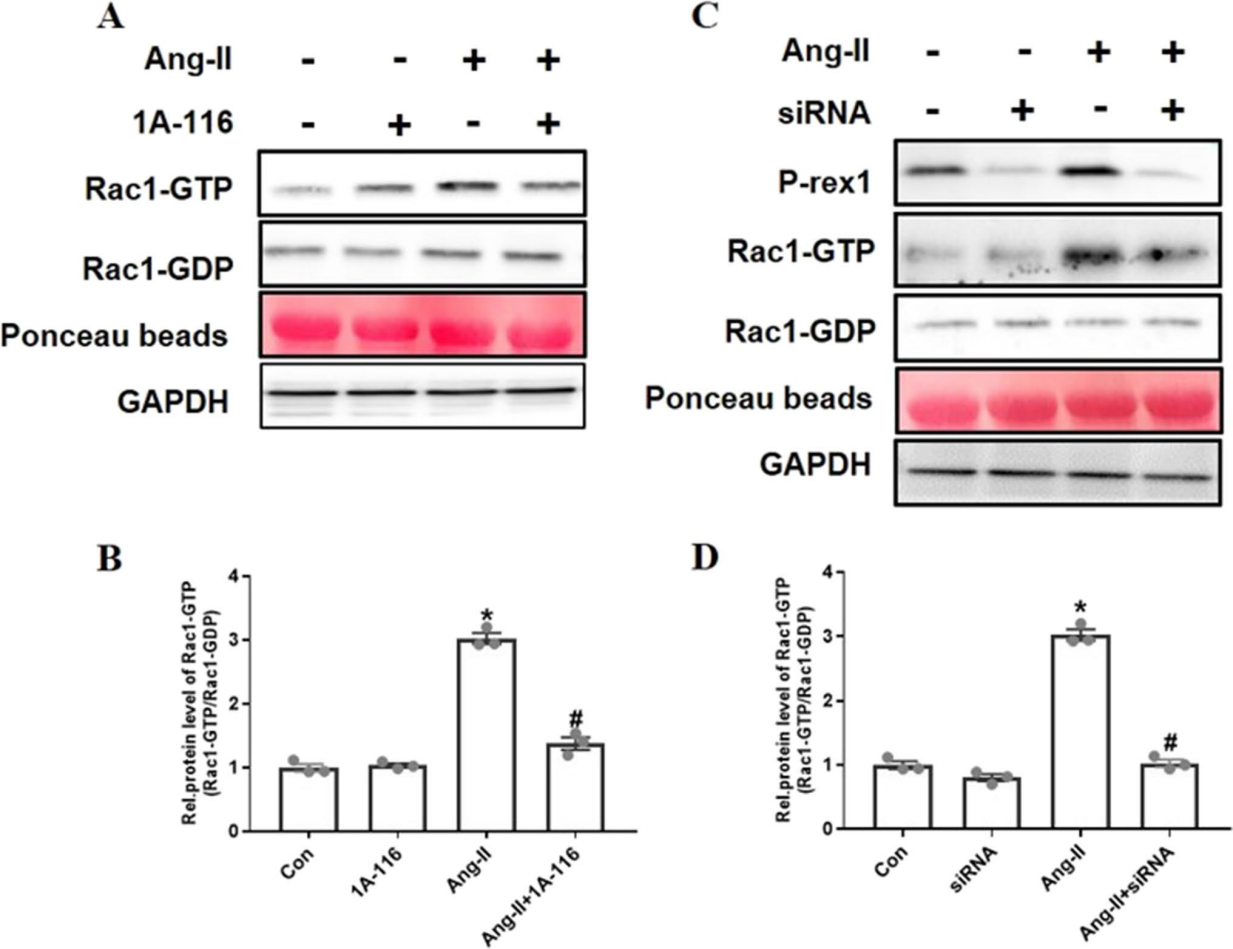
P-Rex1 is an essential mediator of Rac1 activation in response to AngII.** A** Inhibition of P-Rex1 activity in NRCFs impaired AngII-induced Rac1 activation. Serum starved NRCFs were pre-treated with 30 μM 1A-116 for 30 min and then were stimulated with AngII (1 μM, 90 s). The fraction of Rac1-GTP was isolated by pulldown. Both GAPDH and Ponceau staining were used as loading controls. **B** Densitometric values of Rac-GTP levels (normalized to Rac1-GDP) are presented as mean±SD (*n* = 4). ^*^P < 0.05 versus control group, ^#^*P* < 0.05 versus AngII treatment group. **C** Stable depletion of P-Rex1 in NRCFs impairs AngII-induced Rac1 activation. NRCFs were transfected with validated P-Rex1 siRNA or control duplexes. After 24 h, cells were serum starved for 24 h and then stimulated with AngII (1 μM, 90 s). The fraction of Rac1-GTP was isolated by pulldown. Both GAPDH and Ponceau staining were used as loading controls. **D**Fold induction in Rac1-GTP levels normalized to Rac1-GDP, as determined by densitometry, is expressed as mean±SD (*n* = 4). ^*^P < 0.05 versus control group, ^#^*P* < 0.05 versus AngII treatment group

AngII-induced Rac1 activation has been proven to play a critical role in the procession of cardiac fibrosis by promoting the secretion of CTGF [9, 14]. Here, we detected whether P-Rex1 could mediate Rac1 activation in response to AngII stimulation in CFs. As a first approach to address this issue, we used a P-Rex1 specific inhibitor 1A-116 to pre-treat starved CFs for 40 min and blocked P-Rex1 activity. The fraction of Rac1-GTP was isolated in virus-transfected cells by GST-pulldown-assay [15]. Blocking P-Rex1 activity with 1A-116 decreased AngII-induced activation of Rac1-GTP (fold reduction compared with AngII, 0.58±0.05, ^*^ *P*< 0.05) (Fig. 2A and B). Another approach was to use P-Rex1–specific siRNA to transfect CFs. Depletion of P-Rex1 expression also showed a significant reduction of AngII-induced activation of Rac1-GTP (fold reduction compared with AngII, 0.52±0.04, *P* < 0.05) (Fig. 2C and D). Taken together, these results indicated that P-Rex1 was a key regulator in AngII-driven Rac1 activation.

### P-Rex1 Couples with AngII-Induced Activation of Pak1/2/3, ERK1/2

**Fig. 3:**
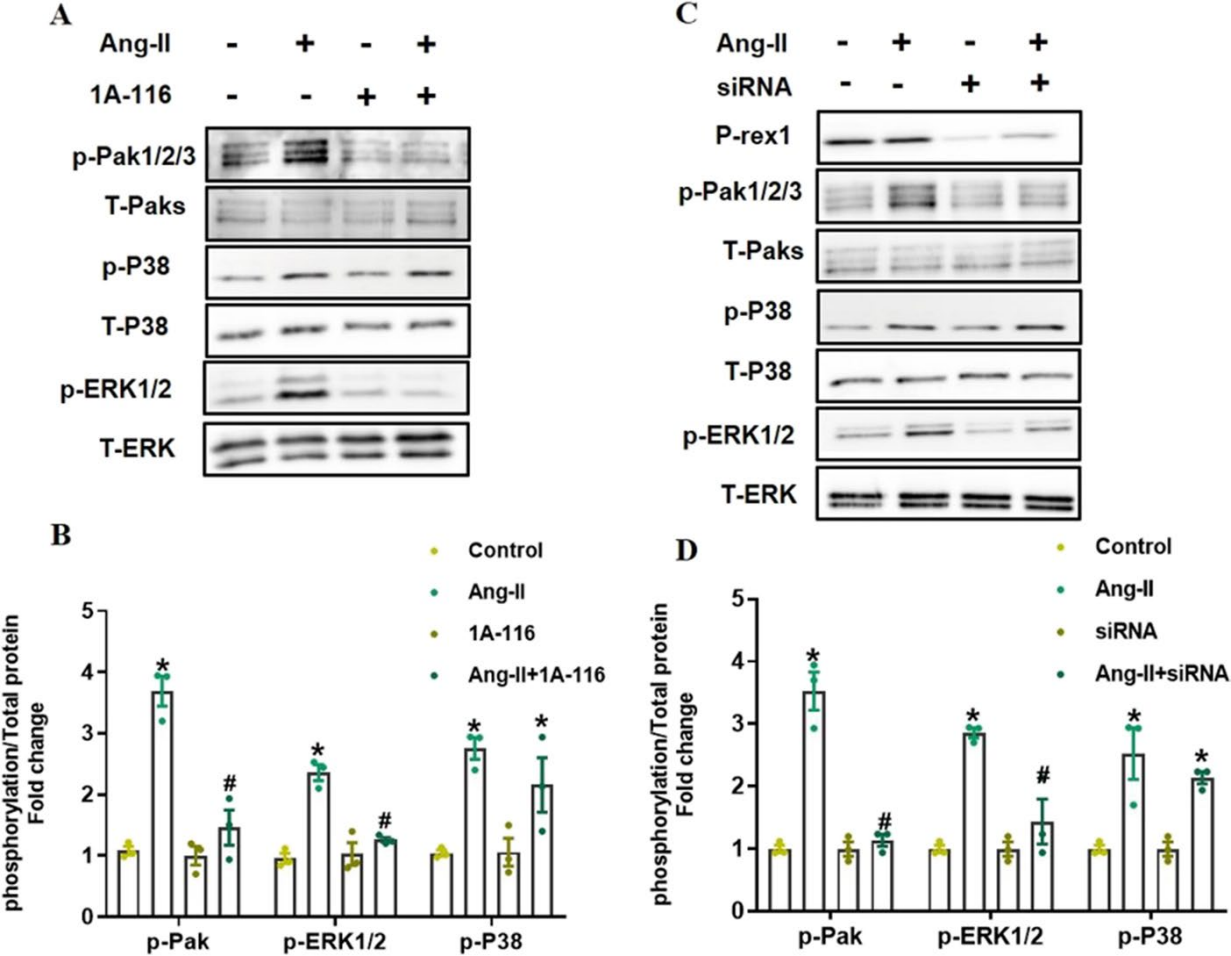
P-Rex1 regulates AngII-induced activation of Rac1 downstream effector Pak1/2/3 and ERK1/2.** A** Inhibition of P-Rex1 activity in NRCFs impairs AngII-induced phosphorylation of Pak1/2/3 and ERK1/2, but not P38. Serum starved NRCFs were pre-treated with 30 μM 1A-116 for 30 min, and then were stimulated with AngII (1 μM, 5min). Total Paks, total ERK1/2, and total P38 were used as loading controls. **B** Quantification of western blotting data by densitometry. Fold change is indicative of ratio of phosphorylated Pak1/2/3, ERK1/2, and P38 to total Pak1/2/3, ERK1/2, and P38, respecticely. Shown are mean±SD of relative density based on 4 experiments. ^*^P < 0.05 versus control group, ^#^*P* < 0.05 versus AngII treatment group. **C** Stable depletion of P-Rex1 in NRCFs impairs AngII-induced phosphorylation of Pak1/2/3 and ERK1/2, but not P38. NRCFs were transfected with validated P-Rex1 siRNA. After 24 h, cells were serum starved for 24 h and then stimulated with AngII (1 μM, 5 min). Total Paks, total ERK1/2, and total P38 were used as loading controls. **D** Quantification of western blotting data by densitometry. Fold change is indicative of ratio of phosphorylated Pak1/2/3, ERK1/2, and P38 to total Pak1/2/3, ERK1/2, and P38,respecticely. Shown are mean±SD of relative density based on 4 experiments. ^*^P < 0.05 versus control group, ^#^*P* < 0.05 versus AngII treatment group

Pak1/2/3 [8] is one of the best-characterized effectors of Rac1 and could be phosphorylated at different phosphorylation sites [16]. Past studies suggested that Pak1/2/3 was a mediator of fibrosis in other tissues such as the liver and kidney [17]. In this study, we explored whether P-Rex1 could regulate the phosphorylation of Pak1/2/3 in response to AngII stimulation. Inhibition of P-Rex1 activity with 1A-116 attenuated the AngII-induced phosphorylation of Pak1/2/3 (fold reduction compared with AngII, 0.71±0.03, *P* < 0.05) (Fig. 3A and B). Transient depletion of P-Rex1 with siRNA also showed a significant reduction in the AngII-induced phosphorylation of Pak1/2/3 (fold reduction compared with AngII, 0.68±0.02, *P* < 0.05) (Fig. 3C and D).

In cardiac fibrosis, the rapid activation of MAP kinases in response to RAAS and SNS activation has been well documented [18]. Activation of MAP kinases has also been proved in CFs subjected to AngII stimulation. Here, we determined whether P-Rex1 may couple with AngII-driven phosphorylation of P38 and ERK1/2. Blocking P-Rex1 activity with 1A-116 decreased AngII-driven activation of ERK1/2 (fold reduction compared with AngII, 0.54±0.04, *P* < 0.05) (Fig. 3A and B), but phosphorylation of P38 without any change. Also, ERK1/2 phosphorylation was blunted by depletion of P-Rex1 expression with siRNA (fold reduction compared with AngII, 0.42±0.05, *P* < 0.05) (Fig. 3C and D). Conversely, p-P38 remained unaffected.

The data obtained in these phosphorylation studies suggest that the AngII-induced P-Rex1/Rac1 activation could regulate downstream effectors such as the profibrotic transcriptional regulator Pak1/2/3 and ERK1/2.

### Inhibition of P-Rex1 Mediate AngII-Induced Oxidative Stress

Past studies have demonstrated that oxidative stress showed a critical role in AngII-induced cardiac fibrosis, especially in AngII activated ROS-sensitive kinases and promoted ROS generation [19]. In our present study, Nox activity level was increased in the cardiac fibroblasts after AngII stimulation, but P-Rex1 specific inhibitor 1A-116 and siRNA could attenuate this stimulus effect (Supplementary Fig. 1A and B). The upregulation level of superoxide anion induced by AngII was inhibited by blocking P-Rex1 (Supplementary Fig. 1C and D). AngII induced the promotion of MDA production and also was blocked by the 1A-116 and siRNA treatment in CFs (Supplementary Fig. 1E and F). SOD activity was inhibited by AngII, while blocked P-Rex1 administration reduced the decrease of SOD activity (Supplementary Fig. 1G and H). The above results all indicate that AngII-induced oxidative stress could be effectively suppressed via P-Rex1 specific inhibitor 1A-116 or siRNA.

### P-Rex1 Mediate AngII-Induced Fibrotic Responses

**Fig. 4:**
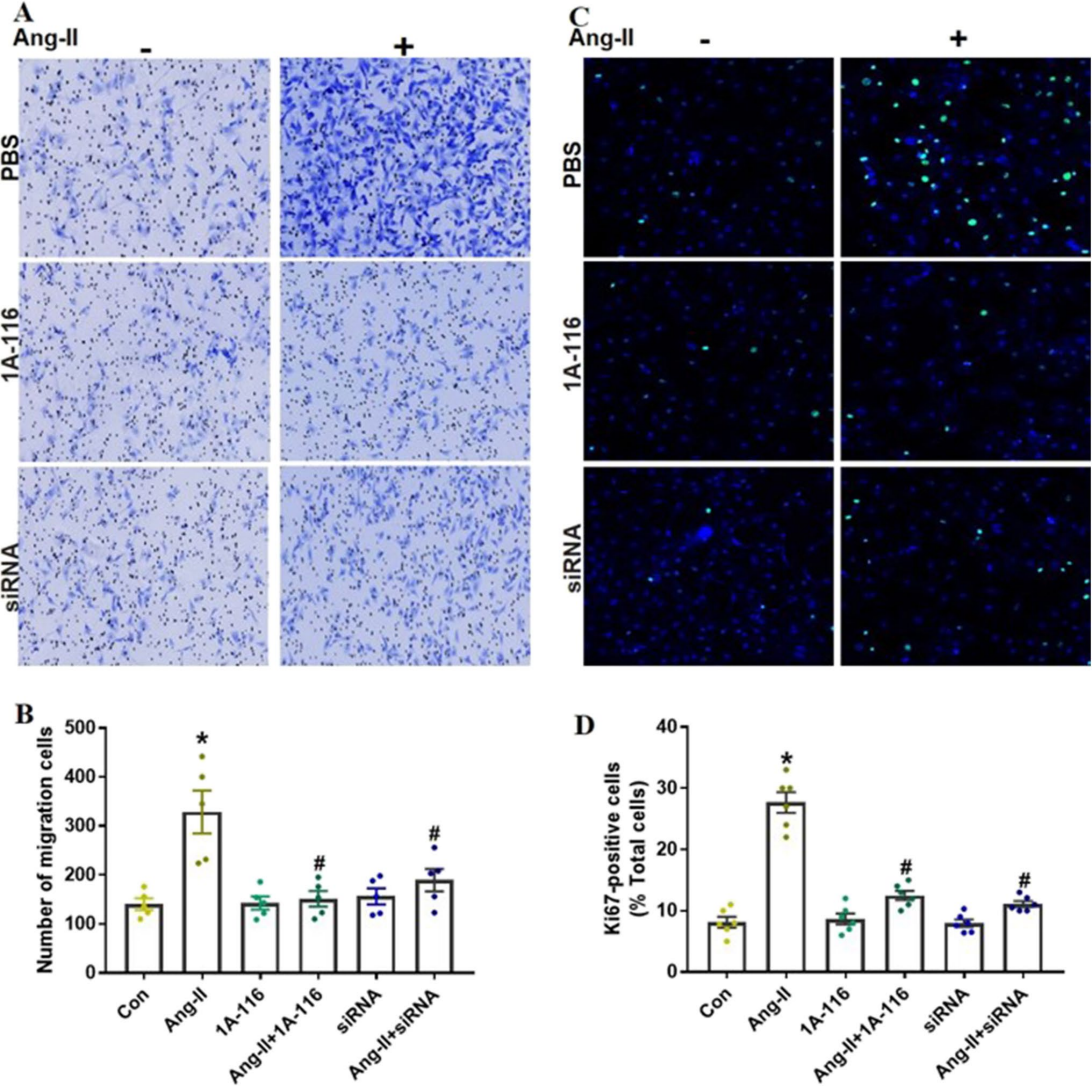
P-Rex1 mediate AngII-induced fibrotic responses in CFs. CFs treated with P-Rex1 specific inhibitor (1A-116) or transfected with P-Rex1 specific siRNA. Cells were further stimulated with or without 100 nM AngII for 24 h. **A/B** Transwell migration assay was used to test the cell migration ability. Representative images from the transwell migration assay and quantification of migrated fibroblasts of the indicated groups. All data are presented as the mean±SD. ^*^P < 0.05 versus control group, ^#^*P* < 0.05 versus 1A-116 treatment group or siRNA group, *n* = 4. **C/D** The proliferation of CFs was measured by ki67 incorporation assay. Representative images from the ki67 staining assay and quantification of proliferated fibroblasts of the indicated groups. All data are presented as the mean±SD. ^*^P < 0.05 versus control group, ^#^*P* < 0.05 versus 1A-116 treatment group or siRNA group, *n* = 4

CFs migration, transdifferentiation, and proliferation were considered the key cellular events in response to myocardial infarction (MI) [3], myocarditis [4], or volume overload [5]. Past studies have proved that P-Rex1 plays a critical role in the invasion, migration, and proliferation of tumor cells or endothelial cells [13, 20]. In this study, to evaluate the P-Rex1 role in AngII-mediated increases in CFs migration, we determined cell viability via trans-well migration assay. CFs were divided into four groups and treated according to the method described above. Our data showed that inhibition or depletion of P-Rex1 abrogated the increased cell migration induced by AngII stimulation (Fig. 4A and B). Consistent with these findings, Ki67 staining data suggested that AngII-induced proliferation was markedly attenuated by blocking P-Rex1 (Fig. 4C and D). P-Rex1 specific inhibitor or siRNA could effectively suppress the proliferation when compared with the AngII group. In summary, these in vitro results demonstrated that inhibition of P-Rex1 could prevent cardiac fibroblast activation and proliferation in response to AngII in vitro.

### Blocking P-Rex1 Attenuated AngII-induced Cardiac Fibrosis In Vitro

**Fig. 5:**
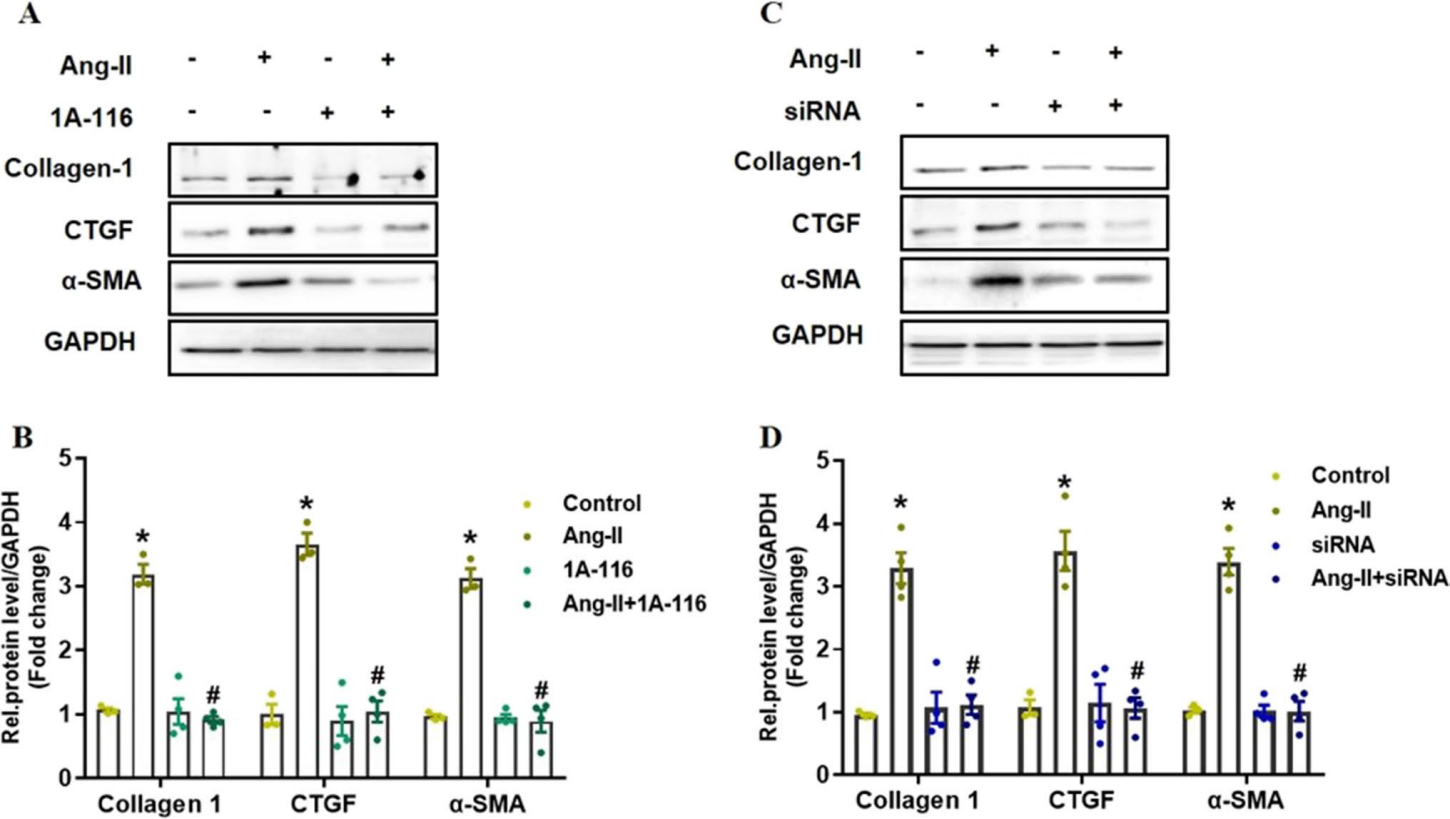
Blocking P-Rex1 on the expression of AngII-induced collagen1, CTGF, and α-SMA expression in vitro.** A** Inhibition of P-Rex1 activity in NRCFs impairs AngII-induced collagen1, CTGF, and α-SMA expression. Serum starved NRCFs were pre-treated with 30 μM 1A-116 for 30 min, and then were stimulated with AngII (100 nM, 24 h). GAPDH was used as loading control. **B** Quantification of western blotting data by densitometry. Fold change is indicative of ratio of collagen1, CTGF, and α-SMA to GAPDH. Shown are mean±SD of relative density based on 4 experiments. ^*^P < 0.05 versus control group, ^#^*P* < 0.05 versus AngII treatment group. **C** Stable depletion of P-Rex1 activity in NRCFs impairs AngII-induced collagen1, CTGF, and α-SMA expression. NRCFs were transfected with validated P-Rex1 siRNA. After 24 h, cells were serum starved for 24 h and then stimulated with AngII (100 nM, 24 h). GAPDH was used as loading control. **D** Quantification of western blotting data by densitometry. Fold change is indicative of ratio of collagen1, CTGF, and α-SMA to GAPDH. Shown are mean±SD of relative density based on 4 experiments. ^*^P < 0.05 versus control group, ^#^*P* < 0.05 versus AngII treatment group

Past studies showed that α-smooth muscle actin (α-SMA), collagen1, and CTGF were the hallmark of cardiac fibrosis following the AngII administration [21, 22]. Since P-Rex1 was a regulator of AngII-induced activation of Rac1, we next confirmed the blocking effect of P-Rex1 on AngII-induced cardiac fibrosis in vitro. NRCFs were pre-incubated with or without P-Rex1 specific inhibitor (1A-116) for 30 min, and then stimulated with AngII (100 nM for 24 h). Western blot results and analysis showed a significant up-regulation of fibrotic markers after stimulation with AngII, such as collagen1, CTGF, and α-SMA expression. As expected, those fibrotic markers’ expression was completely prevented by pre-incubation with the small molecule inhibitor (1A-116) (Fig. 5A and B). Those experiments were repeated with another approach to depletion of (P-Rex1 specific siRNA) P-Rex1. Similar to the inhibitor effects, western blot data showed a similar down-regulation of collagen1, CTGF, and α-SMA expression after NRCFs transfected with siRNA plus AngII stimulation (Fig. 5C and D). These results suggested that P-Rex1 was sufficient to promote cardiac fibrosis, and blocking P-Rex1 regulates the cardiac fibrotic collagen expression in vitro.

### Inhibition of P-Rex1 Attenuated Blood Pressure and Cardiac Remodeling in Response to AngII In Vivo

**Fig. 6:**
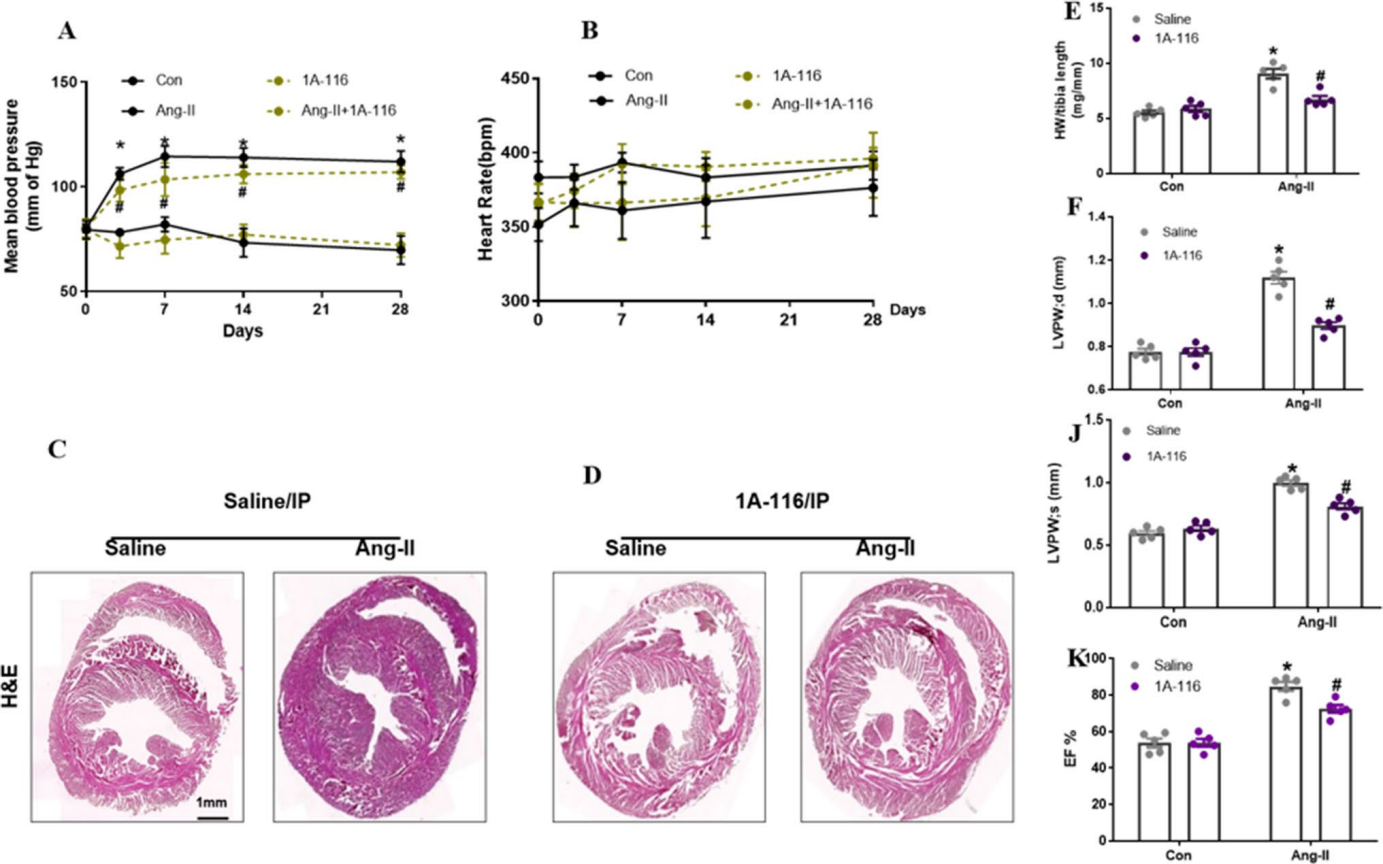
Inhibition of P-Rex1 attenuated blood pressure and cardiac remodeling in response to AngII in vivo.** A** Tail-cuff measurement of mean blood pressure of mice with DMSO or 1A-116 treatment in response to chronic perfusion of saline or AngII administered via osmotic minipumps (*n* = 6). **B** Tail-cuff measurement of mean blood pressure of mice with AngII or AngII+1A-116 treatment (*n* = 6). **C/D** The hearts of mice subjected to Ang-were enlarged on gross morphology. Left, Hematoxylin-eosin (H&E) staining was performed to assess AngII-induced cardiac remodeling between sham and AngII treated mice with saline or 1A-116. **E/F** Echocardiographic parameters were recorded and compared between sham and AngII treated mice with saline or 1A-116 (*n* = 6). **J** Statistical differences in the heart weight/body weight (HW/BW) ratios between sham and AngII treated mice with saline or 1A-116 (*n* = 6). **K** Left ventricular ejection fraction between sham and AngII treated mice with saline or 1A-116 (*n* = 6). All data are presented as the mean±SD. ^*^P < 0.05 vs. the normal saline (control) group, #^#^*P* < 0.05 vs. the AngII treatment group, ^$^*P* < 0.05 vs. the AngII+1A-116 treatment group

To examine the hypothesis that inhibition of P-Rex1 reduces AngII-induced cardiac fibrosis procession in vivo, we generated a control group through saline or AngII chronically perfused into C57BL6/J mice for 4 weeks. In another treatment group, 1A-116 was injected intraperitoneally at 5 mg/kg. Blood pressure, heart rate, and the structure of the heart were monitored through tail-cuff plethysmography and echocardiography. First, we did not find any difference in the baseline of blood pressure between the control group and the 1A-116 treatment group (as shown in Fig. 6A). Plus, the AngII-induced significant increase in blood pressure was observed in both the AngII group and AngII plus 1A-116 treatment group. Interestingly, the AngII-induced increase in blood pressure was significantly attenuated by 1A-116 treatment on day 7 (Fig. 6A). Notably, four groups of mice maintained a similar heart rate during all the study stages (Fig. 6B). Combined, these results indicated that the P-Rex1 inhibitor did not have any influence on the sympathetic nervous system(SNS).

**Fig. 7:**
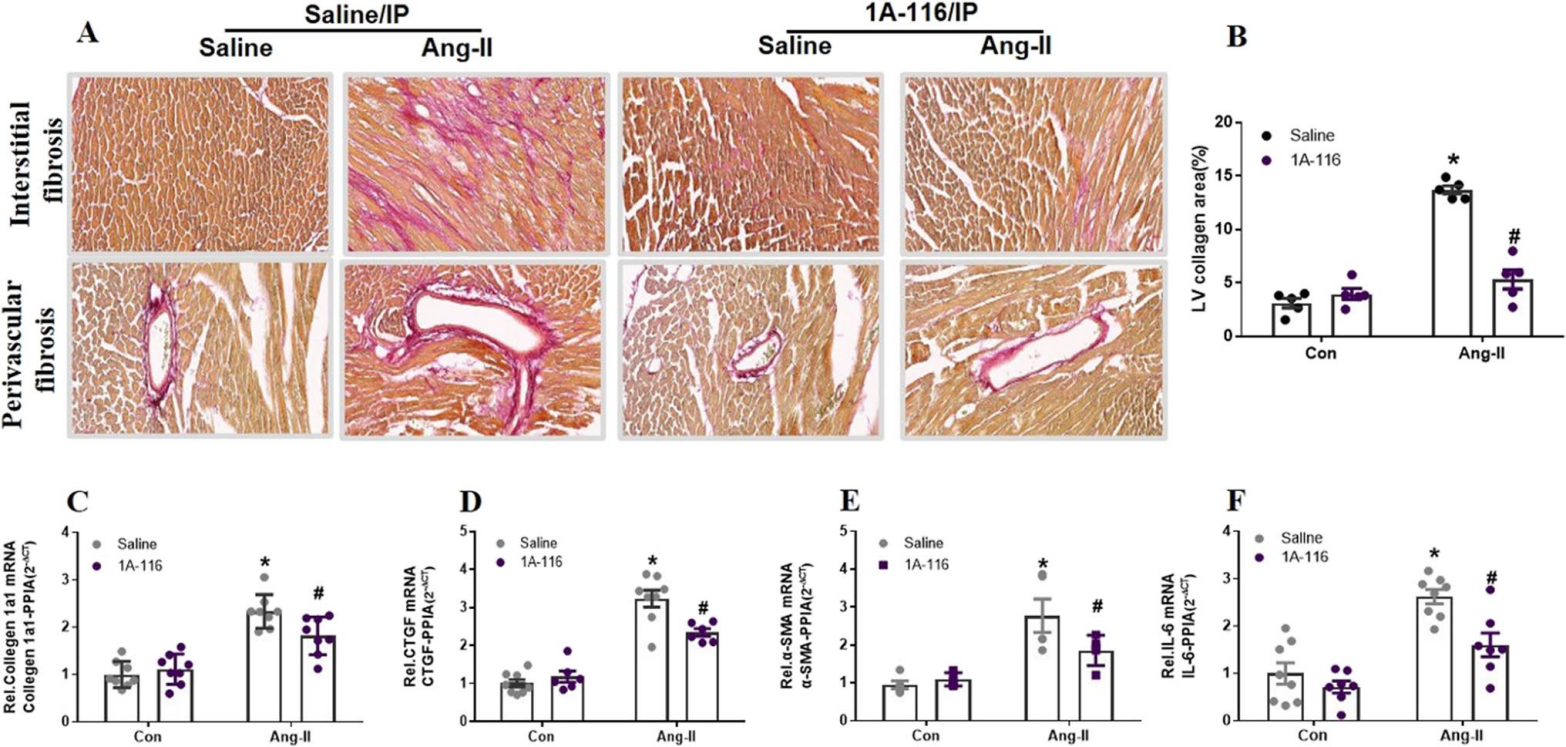
Inhibition of P-Rex1 downregulates AngII-induced cardiac fibrosis in mice. C57BL/6 mice received AngII perfusion (1 mg·kg−1·d−1 ) with or without 1A-116 for 28 days. **A/B** Sirius red staining of histologic sections of LV was performed to assess AngII-induced cardiac remodeling between sham and AngII treated mice with saline or 1A-116. Statistical analysis of difference in cardiac fibrosis (*n* = 6). **C/D/E/F** Quantitative real-time PCR (qRT-PCR) was performed to analyze mRNA levels of fibrotic markers (Collagen1a, CTGF, α-SMA, and IL6,). All data are presented as the mean±SD. ^*^P < 0.05 vs. the normal saline (control) group, ^#^*P* < 0.05 vs. the AngII treatment group

Morphological and histological examinations showed that the inhibition of P-Rex1 attenuated the enlargement of heart size and the increase of HW/BW ratios stimulated by AngII (Fig. 6C, D, and E). On echocardiography, AngII-induced mice plus 1A-116 treatment group showed less increase in LVAWs, LVAW_d_, and LVEF compared with the AngII-induced group (Fig. 6F, J, and K). Taken together, treatment with P-Rex1 inhibitor ameliorated AngII-induced abnormalities in heart structure and function. AngII-induced cardiac fibrosis and collagen deposition were significantly decreased after 1A-116 treatment in LV tissues, particularly in the interstitial and perivascular areas (Fig. 7A and B). Consistent with the results used for in vitro tests, in AngII-infused mice, the increased mRNA levels of fibrotic factors, such as collagen I, CTGF, α-SMA, and IL-6 were all attenuated by P-Rex1 specific inhibitor 1A-116 (Fig. 7C–F). The above data demonstrated that blocking P-Rex1 by its specific inhibitor could protect mice from AngII-induced cardiac fibrosis, and suggested that P-Rex1/Rac1/Paks cascade had therapeutic potential in cardiac remodeling.

## Discussion

**Fig. 8:**
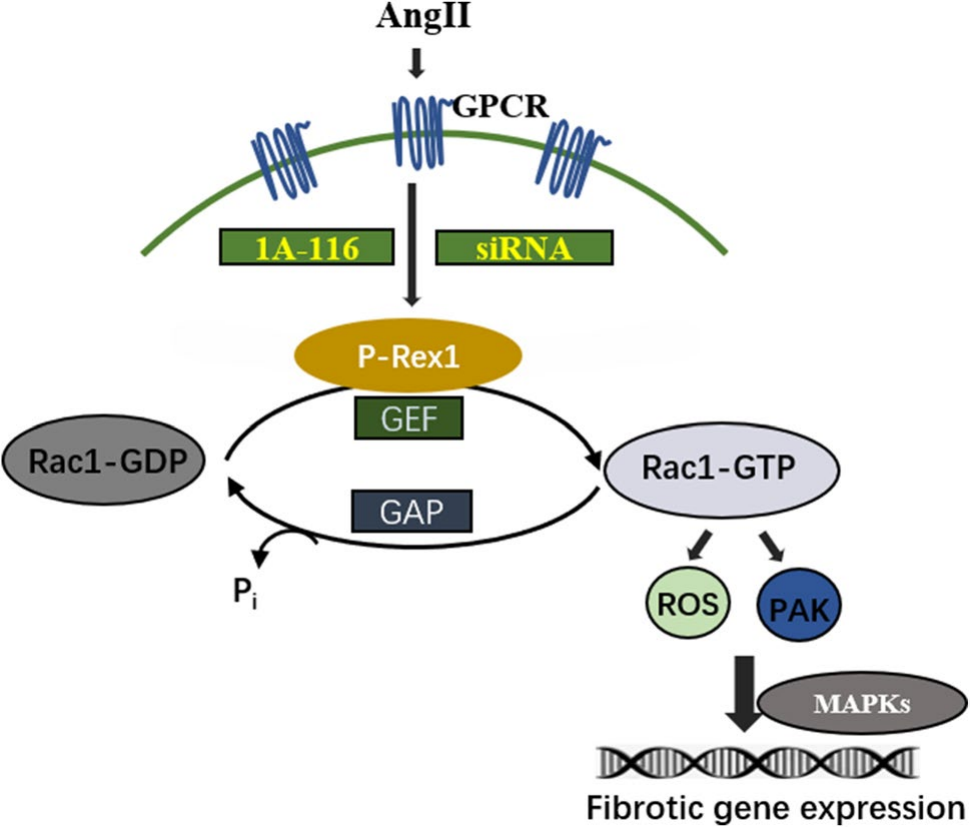
Schematic model showing the involvement of phosphatidylinositol (3,4,5)-trisphosphate–dependent Rac exchanger 1 (P-Rex1) in angiotensin II–induced cardiac fibrosis. See the Discussion section for a description of the role of P-Rex1 in AngII–induced Rac1 activation, Paks phosphorylation, ERK1/2 activation, reactive oxygen species (ROS) production, and collagen generation

A sudden loss or death of massive cardiomyocytes following different kinds of pathological conditions overwhelms the limited regenerative capacity of the myocardium, leading to cardiac fibrosis or hypertrophy, and finally increased mortality in cardiovascular diseases [23]. CFs activation was the early step of cardiac fibrosis and could be stimulated by mechanical and neurohumoral stimuli, such as the RAAS and SNS. Thus, research on the understanding of the pathogenesis and mechanism of cardiac fibrosis was of great importance and could identify promising targets for the prevention and treatment of the patient with cardiac fibrosis. To the best of our knowledge, these findings for the first time found that P-Rex1 was an essential mediator of CFs activation in vitro and subsequent cardiac fibrosis in vivo, and 1A-116 could be a potential pharmacological development target (Fig. 8).

Rac protein, one of the members of the Rho protein family, has three distinct isoforms: Rac1, Rac2, and Rac3. Recently, evidence had revealed that Rac1 activation plays a critical role in CTGF expression, production of ECM, and cardiac fibrosis [24]. In the present study, Rac1-GTP was significantly up-regulated under AngII stimulation; conversely, AngII-induced activation of Rac1 was inhibited by blocking P-Rex1. These data indicate that P-Rex1 exerts its GEF function and an anti-fibrotic activation property through the inhibition of Rac1-GTP activation.

GTP-bound Rac1 binds the regulatory domain of Paks, which leads to a conformational change and removes the trans-inhibitory switch, therefore allowing its autophosphorylation [25]. Past studies have demonstrated that Pak kinases play a key role in biological processes such as cell migration, survival, and proliferation [8]. Paks have multiple downstream targets, including ERK1/2, p38, Akt, and PP2A, which have been shown to interact with Pak proteins [26], thereby contributing to the activity of transcriptional factors such as NFkB, SRF, MEF2, and AP-1 [27]. In line with previous reports, we also found AngII can activate Pak1/2/3, ERK1/2, and p38 phosphorylation, and blocked P-Rex1 could markedly inhibit the activation of Pak1/2/3 and ERK1/2 in vitro. Therefore, we believe that P-Rex1 plays a critical role in inhibiting cardiac fibrosis by blocking Paks phosphorylation and MAPK signaling.

The Noxs drive ROS generation and regulate the redox status and various redox-sensitive signal transduction pathways, which play a critical role not only in physiological conditions but also in cardiac fibroblast proliferation and collagen synthesis [28]. Past studies have shown that Rac1 was a subunit of the NOX complex and essential for the activation of NOX1 and NOX2 [29]. Past research has demonstrated that inhibition of NOX activity could effectively block the ROS generation, which was in mitochondria, lipoxygenase, cyclooxygenase, and xanthine oxidase, finally leading to reducing the development of cardiac fibrosis and improving cardiac function [30, 31]. In this study, we observed the reduction of SOD levels in CFs by AngII stimulation, which also was inhibited with P-Rex1 antagomir. Taken together, it appears that blocking P-Rex1 may act as a new approach to preventing cardiac fibrosis through attenuating oxidative stress.

The present study showed that blocking P-Rex1 exerted a protective effect against AngII-induced cardiac fibrosis by inhibiting P-Rex1/Rac1 activation and ROS production, but this study has several limitations. First, blocking P-Rex1 reduced the activation of the Rac1, ROS production, Paks, and ERK pathways induced by angiotensin II. However, we did not use a Paks or ERK inhibitor to detect the effect on cardiac fibrosis and the expression of downstream pro-fibrotic signaling pathways. Second, the P-Rex1 level was upregulated in AngII-induced mouse models. However, we did not observe overexpression of P-Rex1 levels and the changes in Rac1 activation and ROS production in the mechanism experiment. Therefore, whether overexpression of P-Rex1 is directly or indirectly leading to cardiac fibrosis is unknown. Our present study suggested a possible relationship between the upregulation of P-Rex1 levels and cardiac fibrosis. However, there remains a lack of clinical investigations on P-Rex1 expression in HF patients’ cardiac tissues. It is not known whether the same conclusion can be drawn in the setting of cardiac fibrosis caused by stimuli other than AngII, for example, by pressure overload, ischemia, or diabetes.

In conclusion, our present study revealed that Rac-GEF P-Rex1 is an essential mediator in AngII-induced cardiac fibrosis. The expression of P-Rex1 showed a time-dependent upregulation in the hearts of mice that received AngII perfusion. Blocking P-Rex1 protected against AngII-induced fibroblast activation both in vitro and in vivo. These findings add useful information about the biological functions of P-Rex1 in CFs activation and subsequent cardiac fibrosis and suggested that modulation of P-Rex1 could be a potential pharmacological development target.

## Supplementary Information

Supplementary Fig. 1 Inhibition of P-Rex1 Mediated AngII-induced Oxidative Stress. **A/B** Nox activity level was increased in CFs after AngII stimulation, but blocked by inhibition of P-Rex1. **C/D** The upregulation level of superoxide anion induced by AngII was inhibited after blocking P-Rex1. **E/F** AngII-induced promotion of MDA production was blocked by the 1A-116 and siRNA treatment. **G/H** SOD activity was inhibited by AngII, while blocked P-Rex1 administration reduced the decrease of SOD activity. Shown are mean±SD of relative data based on 4 experiments. ^*^P < 0.05 versus control group, #^#^*P* < 0.05 versus AngII treatment group. (PNG 468 kb)

High resolution image (TIF 332 kb)

## Data Availability

All the data, material, and statistical code in this study are available upon reasonable request from the corresponding author.
