# Supplementary figures and images for: Pharmacological Inhibition of P-Rex1/Rac1 Axis Blocked Angiotensin II-Induced Cardiac Fibrosis
Source: Cardiovasc Drugs Ther. 2023 Mar 9;38(5):861–72. doi: 10.1007/s10557-023-07442-3 (PMC11438833; doi:10.1007/s10557-023-07442-3)

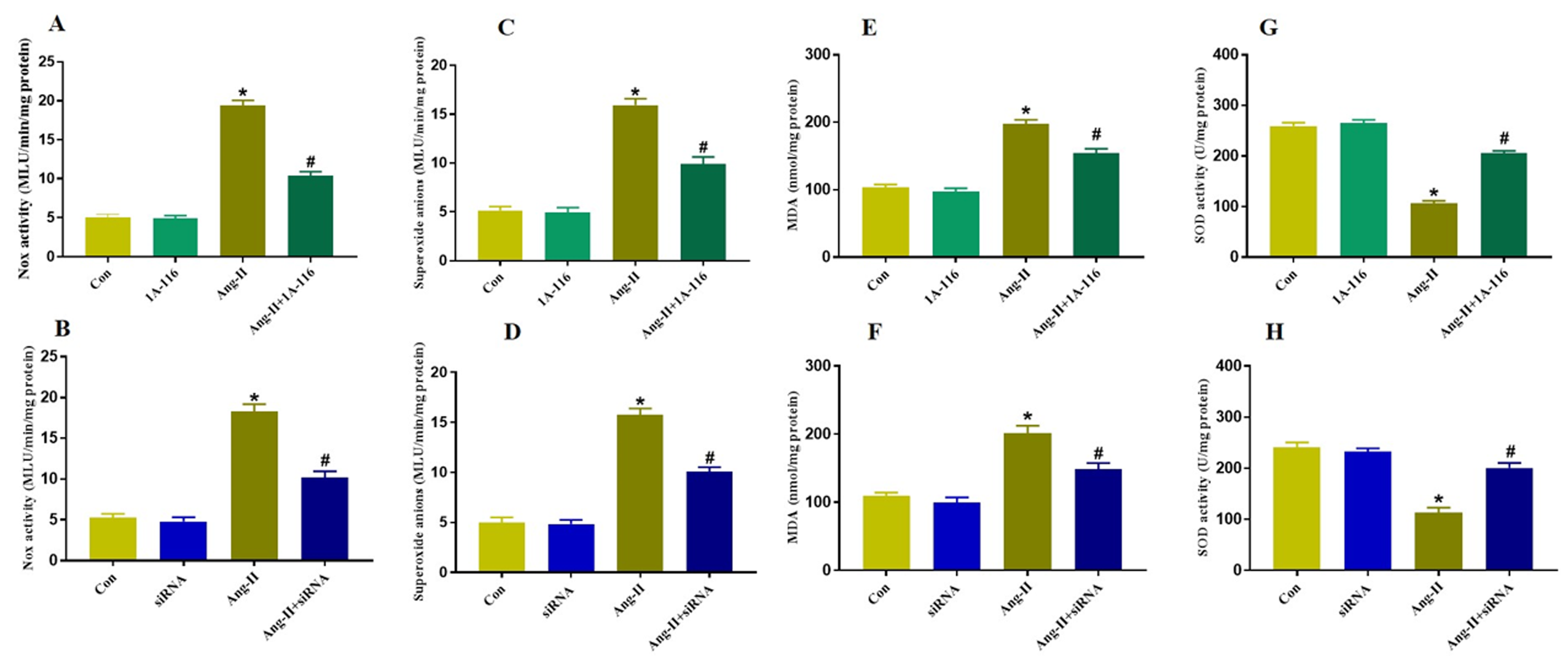

Supplement: Supplementary file 1 — Inhibition of P-Rex1 Mediated AngII-induced Oxidative Stress. A/B Nox activity level was increased in CFs after AngII stimulation, but blocked by inhibition of P-Rex1. C/D The upregulation level of superoxide anion induced by AngII was inhibited after blocking P-Rex1. E/F AngII-induced promotion of MDA production was blocked by the 1A-116 and siRNA treatment. G/H SOD activity was inhibited by AngII, while blocked P-Rex1 administration reduced the decrease of SOD activity. Shown are mean±SD of relative data based on 4 experiments. *P < 0.05 versus control group, ##P < 0.05 versus AngII treatment group. (PNG 468 kb) [file 10557_2023_7442_Fig9_ESM.png]

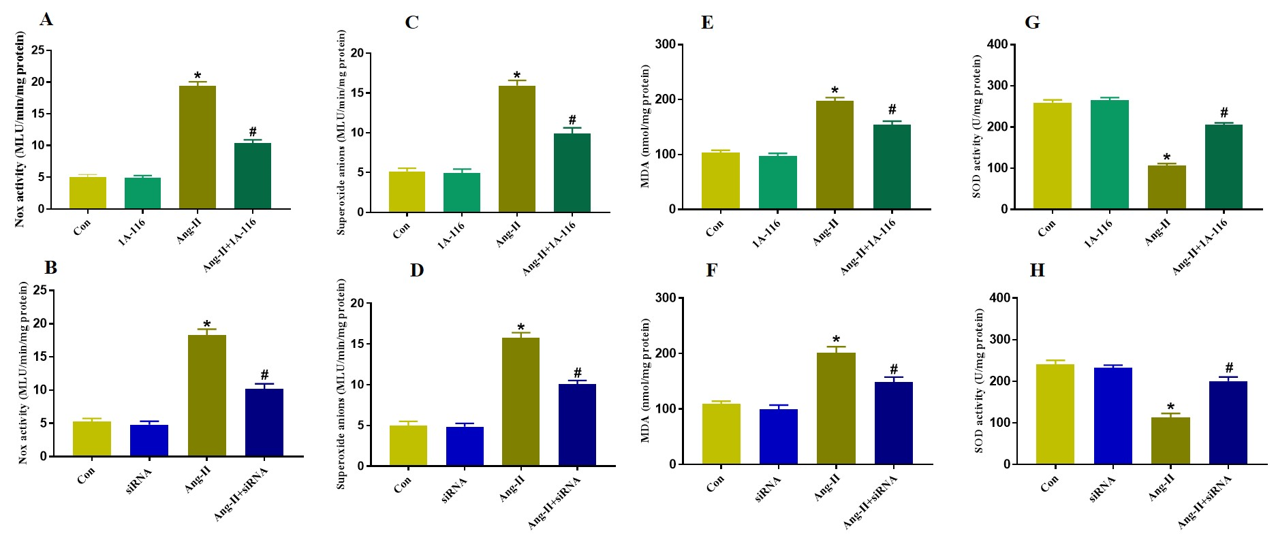

Supplement: Supplementary file 2 — High resolution image (TIF 332 kb) [file 10557_2023_7442_MOESM1_ESM.tif]
